# A Dual-Connectivity Mobility Link Service for Producer Mobility in the Named Data Networking

**DOI:** 10.3390/s20174859

**Published:** 2020-08-27

**Authors:** Ju-Ho Choi, Jung-Hwan Cha, Youn-Hee Han, Sung-Gi Min

**Affiliations:** 1 Department of Computer Science and Engineering, Korea University, Seoul 136-713, Korea; jubong@korea.ac.kr (J.-H.C.); jockercha@korea.ac.kr (J.-H.C.); 2 School of Computer Science and Engineering, Korea University of Technology and Education, CheonAn 330-708, Korea; yhhan@koreatech.ac.kr

**Keywords:** Named Data Networking (NDN), Information-Centric Networking (ICN), mobility, producer mobility, Cyber-Physical System (CPS), Internet of Thing (IoT)

## Abstract

With the exponential growth of Cyber-Physical Systems (CPSs) technologies, the Internet of Things (IoT) infrastructure has evolved from built-in static infrastructure to a flexible structure applicable to various mobile environments. In this Internet of Mobile Things (IoMT) environment, each IoT device could operate simultaneously as a provider and consumer of information, and could provide new services through the exchange of such information. Named Data Networking (NDN), which could request data by content name rather than location (IP address), is suitable for such mobile IoT environments. However, in the current Named Data Networking (NDN) specification, producer mobility is one of the major problems in need of remedy. Previously proposed schemes for producer mobility use an anchor to hide the producer’s movement from consumers. As a result, they require a special anchor node and a signaling procedure to track the current locations of contents. A few anchorless schemes have also been proposed, but they still require mobility signaling and all NDN routers on the signaling path must understand the meaning of the signaling. We therefore propose an anchorless producer mobility scheme for the NDN. This scheme uses a dual-connectivity strategy that can be expressed as a soft handover. Whenever a producer changes its NDN Access Router (NAR), the new mobility link service located on the mobile producer’s old NDN face repairs the old link so that the connectivity with the pNAR can be maintained for a while. The old NDN face is removed after the new location information on the contents of the producer is disseminated over the NDN network by the Named-data Link State Routing Protocol (NLSR) routing protocol at the nNAR. The new mobility link service decouples connection and transaction to hide the collapse of the link. Therefore, the NDN’s mobility procedure could be simplified as the handover is defined as transaction completion as opposed to a breakdown of links. The proposed scheme prevents the routing information from being abruptly outdated due to producer mobility. Our simulation results show seamless handover when the producer changes its default access router.

## 1. Introduction

The Internet of Things (IoT) environment in which various objects are connected through wired and wireless networks to collect, process, exchange, and share information has become an important technology for building a smart city due to the development of cyber-physical system technologies. CPS collects and analyzes information in the physical world through various smart sensors, and immediately applies the results to the physical world through an actuator, so that the cyber world and the physical world are closely connected and cooperate. At this time, the role of the smart sensor has been expanded to be able to move freely, not just a device placed in a fixed position, and another service could be provided through the mobility of these IoT devices [1]. In this Internet of Mobile Things (IoMT) environment, each IoT device could operate simultaneously as a provider and consumer of information, Named Data Networking (NDN) [2,3], which is a new promising Internet architecture that is currently being used to build a distribution network, is suitable for these IoMT environments [4]. The Internet uses the location-based address and stateless forwarding paradigm, but NDN supports information-centric addresses and the stateful forwarding plane [5]. Consumers do not need to know the locations of the contents in which they are interested, because the consumer requests content by mentioning the content name to an NDN router. The NDN Router is then responsible for forwarding the consumer’s Interests to the node where the content is stored. In contrast to routers for the Internet, NDN routers store some content, and pending content requests are stored in their caches.

However, there are challenges associated with supporting producer mobility [6] in NDN. For example, as a producer randomly moves, it changes its NDN Access Router (NAR) in an unpredictable manner. This causes it to miss the delivery of Interests to the producer. If the producer is the sole owner of the specific content and the content is only stored with the producer, all the Interests for the content should be forwarded to the producer. In order to decide which NDN face should be used to forward an Interest for content to the producer, the NDN router refers to the Forwarding Information Table (FIB). The FIB table is generated by the NLSR routing protocol [7] on the NDN router. If the producer changes its NAR, the NDN router may choose an NDN face to deliver the Interest to a NAR that the producer was connected to in the past. Until the FIB is updated, Interests towards the producer will be delivered to the out-of-date NAR.

There have been several proposals put forth regarding NDN producer mobility. These can be divided into two categories: anchor-based schemes and anchorless schemes. Anchor-based schemes [8,9,10,11,12] introduce an anchor (or a rendezvous server) to track the current locations of contents. The anchor stores the binding information for content and its location. The anchor usually interacts with the mobile producers to maintain updated binding information. Anchor-based schemes resemble the Home Agent, as defined in [13,14]. However, it causes suboptimal routing problems and signaling overhead. In anchorless schemes [15,16], mobile producers send special announcements to the previous NAR whenever they change the point of attachment. Therefore, all NDN routers on the signaling path must understand the meaning of the special announcements. Consequently, the anchorless schemes modify the NDN router architecture so that it can handle both ordinary Interests and Interests that require redirection. This may break the simple architecture principle of NDN [17].

In this paper, we propose an anchorless NDN producer mobility scheme. The proposed scheme neither modifies the current NDN router architecture nor announces the producer’s movement to the NDN nodes. It treats the exchange of the <Interest, Data Packet> pair as a transaction. In this respect, the mobility concept in NDN can be considered to be a disruption of transaction as opposed to a breakdown of links. If transactions could be completed while hiding the collapse of the link, the NDN’s mobility procedure could be simplified. Therefore, the proposed scheme introduces dual connectivity to both hide the link breakage to the NDN system and support producer mobility. This strategy that can be expressed as a soft handover. It defines a new NDN link service called the Producer Mobility Link Service (PMLS). The PMLSs in the old NDN faces hide the link breakage to the NDN system. They repair the old link so that the connectivity with the previous NAR can be maintained for a while and re-synchronize the on-going transmissions, if any exist. In addition, the PMLS at the pNAR buffers the Interests arriving during the breakage period and forwards the buffered Interests to the producer following reconnection. The old NDN face is removed after the new location information on the contents of the producer is disseminated over the NDN network by the Named-data Link State Routing Protocol (NLSR) routing protocol at the new NAR. As a result, no Interests miss being delivered due to the producer’s movement. In other words, the PLMS extends the lifetime of the current routing information until the new routing information is disseminated over the network. Therefore, there is no need to modify the NDN router architecture for the mobility service, and the NDN router architecture and mobility service can evolve independently of each other.

## 2. Named Data Networking

NDN reorganizes the existing Internet communication paradigm based on information. This is a concept that attaches great importance to the information itself, not the place. Instead of designating a location such as an IP address, information is considered to be the best independent subject by giving the information a unique identifier or name. Given that the public Internet is getting more crowded, implementing the Internet that focuses on the information itself increases flexibility. It could optimize the flow of information and eliminate the congestion of the points through which information passes.

In NDN communication, a consumer requests a specific content to a producer through an Interest packet. The producer transmits the content requested by the consumer in the Data packet [2]. Additional data structures such as Content Store (CS), a Pending Interest Table (PIT), and a Forwarding Information Base (FIB) are defined for such a content-based communication. The CS is a local cache for Data packets that have been passed through the NDN node. The PIT records pending Interests that have yet to be served. Each PIT entry aggregates the same Interests it receives and remembers incoming NDN faces so that it can build reverse paths for the Interests for data packet forwarding. The FIB is used to forward Interests toward NDN nodes, which have the corresponding content for the Interests. The reference implementation of NDN has been published [18]. The Named Data Networking Forwarder (NFD) implements the core NDN structure.

The NFD interacts with other components via common interface objects called Faces, which are responsible for connection management. A Face can connect the NFD with local applications as well as other local NDN modules, such as the RIB, or remote Faces located at other NDN nodes. The face consists of two components: link service and transport. The NDN link service is a component of the NDN face, and it is responsible for managing the connections between two NDN nodes. The NDN faces connect NDN nodes and transport Interests and Data packets between them [19]. The faces are generated when a new association is detected between two NDN nodes, and they are removed when the connectivity between two NDN nodes is broken. When the producer changes its point of attachment to a new NAR (nNAR), the current NDN faces connecting the producer with the previous NAR (pNAR) are removed and new NDN faces are created to connect the producer with the nNAR. The producer then registers its content to the nNAR via the new NDN faces. The NLSR routing protocol at the nNAR announces the presence of the contents to the NDN routers on the network. Until new routing information is disseminated over the network, many Interests sent to the producer may not reach the producer. These lost Interests usually arrive at the out-of-date pNAR.

The Routing Information Base (RIB) interacts with the FIB via a local Face to manage the FIB. A Name-based routing protocol such as the NLSR [7] interacts with the RIB to install the routing information taken from other NDN router nodes. The RIB includes the Auto Prefix Propagator (APP), which registers local content prefixes to the remote RIB at the current NAR using command Interests [20]. When the remote RIB receives command Interests, it updates its FIB in the NFD and the local content prefixes in the NLSR to accurately reflect the received content prefixes.

## 3. Related Work

According to [6], producer mobility schemes can be categorized as anchor-based schemes and anchorless schemes. Anchor-based schemes use an anchor to maintain the location information of the mobile producer. The anchor has been referred to as a local controller [8], a content router [9], an immobile anchor [10] and a home repository [11]. Depending on the proposed schemes, the anchor either stores the content, forwards Interests, or fetches the content from the producers. The interaction between the anchor and the producers requires extra signaling overhead and may lead to unnecessary triangle routing problems. As a result, it could limit the advantages of NDN, such as multipath communication and its robustness to single point failure.

In the latter category, the mobile producer takes charge of reporting its movement to the network without an anchor. In [12], the previous NAR (pNAR) buffers incoming Interests until receiving a special Interest from the producer. The pNAR then forwards the buffered Interests to the producer. However, when the intermediate NDN router receives the buffered interests from the pNAR, it may discard the buffered interests to avoid routing loop. This is because the intermediate NDN router already has the PIT entry for the same content name. Therefore, before the FIBs at NDN routers on the forwarding path are updated, pNAR must set the bit in the buffered interests indicating that these interests have been re-transmitted. In the anchorless mobility scheme [15], a Temporary FIB (TFIB) is introduced at each NAR to help keep track of the movement of the producer. In LBMA [16], after moving to a new NAR, the producer notifies the corresponding consumer of its current location information using the modified Interest and Data packets. This causes a dependency between the original NDN forwarding plane and the mobility features. In MAP-ME [21], mobile producers use Interest update messages to update the FIB entries in an attempt to minimize their unreachability. T-Move [22] supports producer mobility by using FIB entry updates and pushing contents to the router nearest the producer. These solutions may break the simple architecture principle of NDN [17] as these solutions require some modifications to the existing parts of the NDN architecture, such as the FIB and packet format of interest message.

There are several proposals [23,24,25] put forth to support producer mobility by using an optimal caching configuration. As these caching methods are determined by the heuristic method, the producer mobility may not be guaranteed. The concept of the heuristic solutions here is similar to the predictive mode of Fast Mobile IPv6 (FMIPv6) [26]. Therefore, it can be said that the heuristic solutions have same disadvantages of the predictive mode of FMIPv6 when the mobile node’s movement is incorrectly predicted. The relative signaling cost could increase as the probability of prediction accuracy becomes inaccurate [27]. In addition, if the mobile node moves into unpredicted domain or the handover is not performed immediately, large packet loss and handover latency may occur [28].

## 4. System Overview

When a consumer wants certain content, it sends an Interest to the NAR to which it is attached. The NAR then searches through the routing information at its FIB to forward the Interest to the producer. The routing information is generated by the NLSR of the reference implementation [16]. When the producer moves its location while the Interest is being forwarded, the routing information for the forwarding path within the FIB of the NDN router becomes out-of-date.

The proposed scheme attempts to extend the period in which the routing information within the FIBs of the NDN routers on the forwarding path is relevant. Out-of-date forwarding paths occur when the link connecting the pNAR with a producer is broken due to the movement of the producer. When the producer attaches to a new NAR (nNAR) after it moves, a new Face is created to connect the producer with the nNAR. This allows the transport module of the new Face to communicate with its network. Therefore, the old Face may reconnect the pNAR using the same transport technology used at the transport module of the nNAR. This restores the connectivity between the pNAR and the producer to avoid invalidation of the forwarding path using the pNAR. The old Face removes itself gracefully after the new forwarding path made using nNAR is disseminated over the NDN network. The proposed scheme introduces PMLS, which is a subtype of the link service defined at the NDN specification. The PLMS conceals the link breakage from the NDN forwarding plane and repairs the broken link without needing to introduce a fixed anchor node.

**Figure 1:**
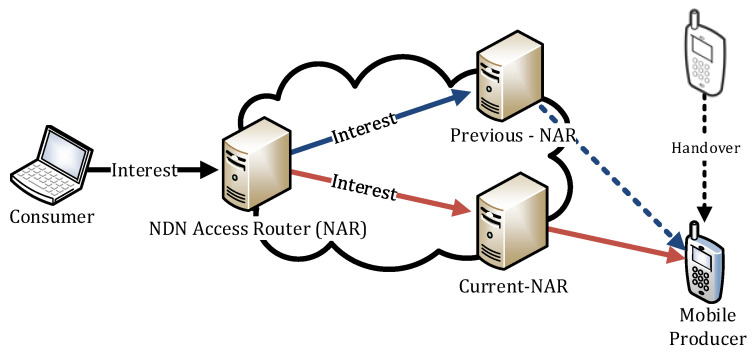
System Architecture Overview.

As shown in Figure 1, only two types of NDN nodes, NDN Mobile Producers (NMPs) and NARs, are involved in the proposed scheme. An NMP is a producer NDN node that stores content and provides it to the consumers requesting the content. It can move randomly. The NMP does not have a name-based routing protocol like NLSR, but its RIB includes an APP module. The NAR is the NDN router at which the NMP is currently attached. The NAR has an NLSR as the name-based routing protocol, and the NLSR is responsible for advertising the content name prefixes registered at the RIB of the NAR. The APP at the NMP delegates the content name announcement process to the NAR. It also propagates the content name prefixes of the contents stored in the NMP to the NLSR via the RIB at the NAR. Both the NMP and the NAR create their own Faces when they are connected, and these Faces use the producer mobility link service as their link service. When the NMP changes its point of attachment from pNAR to nNAR, it maintains dual connectivity: one with pNAR and the other one with nNAR until a new forwarding path using nNAR is stabilized.

Since NDN can run directly over layer 2, Internet Protocol (IP) is not the only layer that should be used as the underlying layer of NDN. Tunneling technology implemented at the data link layer such as Layer 2 Tunneling Protocol (L2TP) can be used as the underlying layer of NDN. However, the proposed scheme assumes that the NDN network employs the IP network as the transport network. It is because that the current IP network is the most popular transport network and it is used at the NDN reference implementation as well.

## 5. A Producer Mobility Link Service in NDN Face

An NDN face consists of a link service and a transport. The generic link service provides fragmentation, reassembly, failure detection, and reliability.

**Figure 2:**
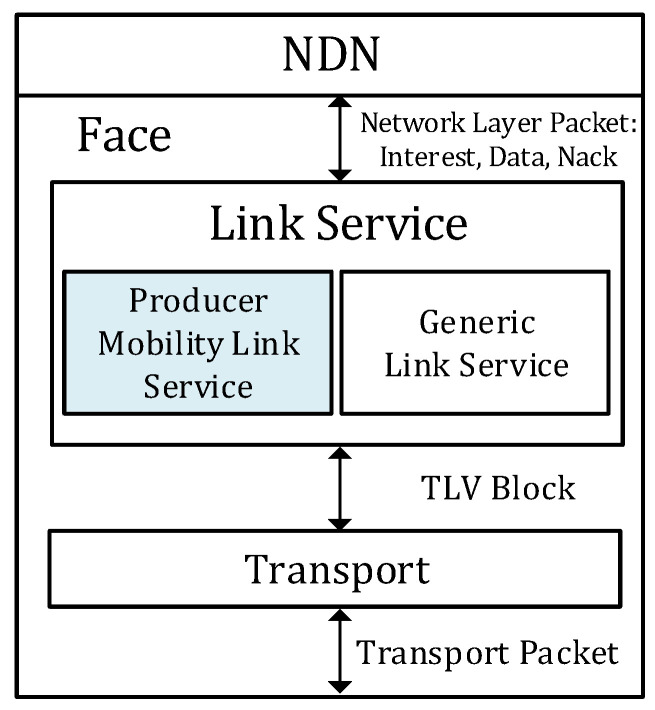
Producer Mobility Link Service in NDN Face.

As shown in Figure 2, the PMLS is added at the link service to conceal the link breakage caused by the movement of the producer. The PMLS supports two additional functionalities: one is the re-establishment of the link and the other is the re-synchronization of the on-going transmission. To re-establish the connection, the PMLS at NAR has a special transport that accepts the connection recovery request from the PMLS at the NMP. When the NDN face of the NMP detects relevant movement, the PMLS sends the Face Update message to the peer PMLS via the special transport at the previous NAR. Then, both PMLSs create new transports to connect to each other.

After re-establishing the connection, the PMLSs must re-synchronize the on-going transmission. If the movement of the NMP occurs when it has received Interest and the corresponding Data packet transmission has yet to be completed, the partially transmitted Data packet is typically discarded. Then, the NMP must wait for the re-transmitted Interest, forcing the consumer to suffer from a long retrieval delay. Therefore, the remaining segments of the partially transmitted Data packet should be transmitted after the connection is fully re-established.

Due to its functionalities mentioned above, the PLMS has the following advantages:

- The PMLS restores links that have been damaged by the producer’s movements so that consumers do not reissue the interests until the default re-transmission delay (3000 ms) expires. After the re-transmission delay expires, it retransmits the interest.
- The PMLS does not change the behavior of the existing NDN unlike the existing schemes that support the mobility of producers by changing the type of FIB or Interest packet.
- As the PMLS supports the mobility of producers by establishing new mobility link services. As it could be inserted and removed like a module in the NDN architecture, it does not require any other modification of the NDN architecture. Therefore, the PMLS which handles the mobility of the NDN has the advantage that it can be advanced regardless of the NDN architecture, and at the same time, the NDN architecture can also evolve regardless of the change of the PLMS.

### 5.1. Transaction Structure

The task unit in NDN can be viewed as the exchange of the <Interest, Data Packet > pair. The PMLS views the exchange of the pair as a transaction.

**Figure 3:**
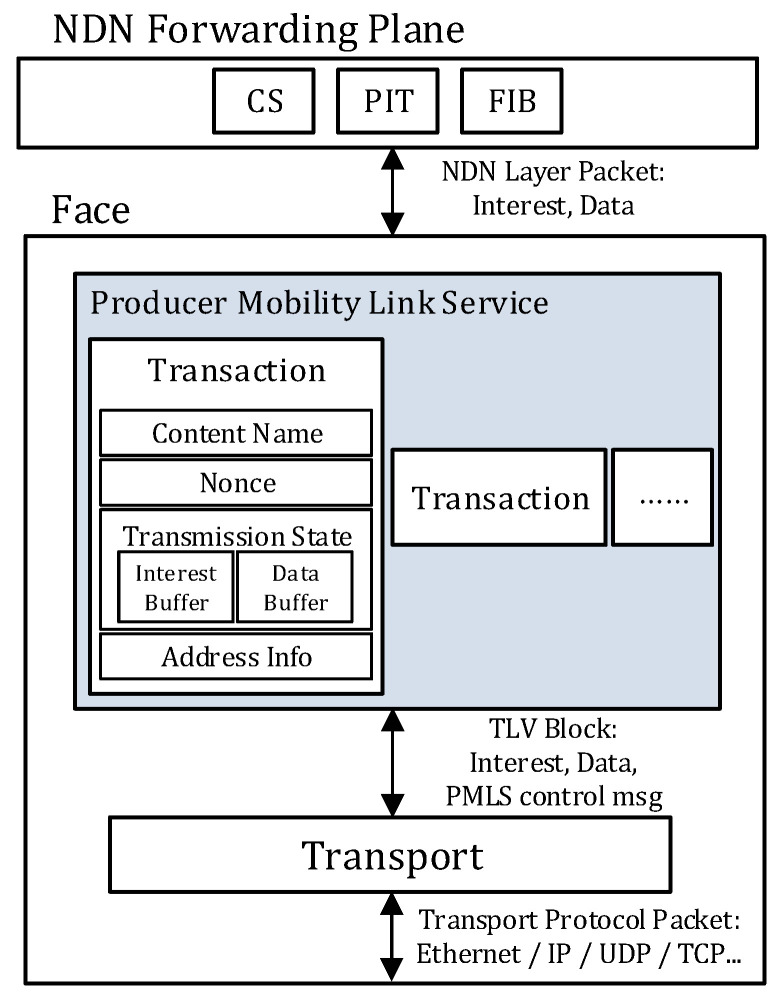
Transaction structure at the Producer Mobility Link Service.

The PMLS defines the transaction structure, which is used to track the exchange state of an <Interest, Data Packet > pair. As an Interest is typically a short message, it is transmitted as a single packet. On the other hand, the Data packet contains a content, so the size of the Data packet may be big enough to be fragmented into segments at the PMLS. When a PMLS receives an Interest from an upper layer or a lower layer, it creates an instance for the transaction structure. Figure 3 shows the transaction structure at the PMLS. The PMLS records the exchange state of the transaction in the instance of the transaction structure and is used to recover the transmission state afterward.

The elements of the transaction structure are defined as follows:

- Content name: The content name derived from the Interest. It is used to identify the instance of the transaction structure in a PMLS.
- Nonce: The fixed-length value from a nonce field of the Interest. The nonce is set by the original data requester. The nonce is included in the PMLS control messages used to identify a relevant instance of the transaction structure at the peer PMLS. It reduces unnecessary packet overhead caused by variable-length content names.
- Transmission state: This is used to track the transmission state of the Data packet segments and to check how much of the expiration time of a transaction remains. A status report message uses this information.
- Address information: The peer transport endpoint information for control messages. It is used to transmit PMLS control messages. It is updated by a status report message sent by its peer PMLS if the state message contains this information.

### 5.2. PMLS Control Message

The PMLS introduces three types of control messages that can be used to recover a broken link: data segments, status reports, and Face update messages. They follow the Type-Length-Value (TLV) structure to meet the common link-layer packet format of the NDN face [18]. They are only used between peer PMLSs.

***Data Segment:*** A Data segment carries a segment of an Interest or Data packet. It includes the segment sequence number.

- Nonce: All data segments belonging to one data packet have the same nonce field of the data packet. This nonce value is directly derived from the nonce value in the received interest packet that uniquely identifies the interest packet.
- Sequence number: It means the relative position of the data segment.
- Last segment flag: It means whether there are more data segments to be sent. In other words, it is set to 1 when it is the last data segment.
- Payload: Partial content of the Data packet.

***Face update:*** A Face update is used to notify a peer PLMS of the new transport endpoint address after the NMP has moved.

- Nonce: The value from the nonce field of an instance of the transaction structure.
- Address information: A new transport endpoint address. It is used to transmit a status report to the peer PMLS.

***Status Report:*** A status report message is used to notify the peer PLMS of the current exchange state of a transaction.

- Nonce: The value from the nonce field of an instance of the transaction structure.
- Missing segment number and length: Content lost due to the movement of the producer.

### 5.3. Handover Operation of PMLS

**Figure 4:**
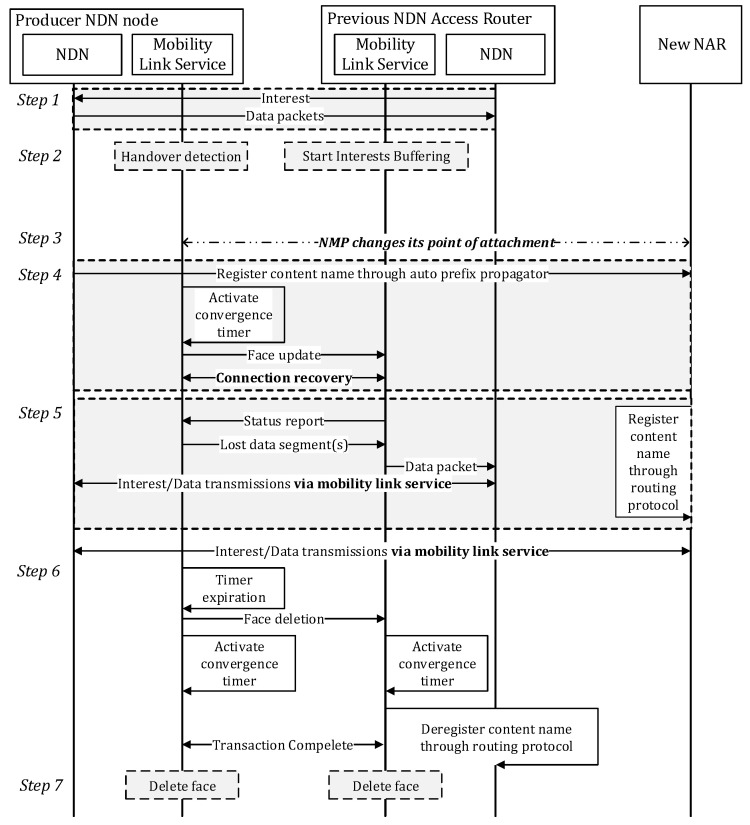
Handover operation of PMLS.

The handover operation of PMLS is illustrated in Figure 4.

***Step 1.*** The NMP exchanges Interests and Data packets with the previous NAR (pNAR).

***Step 2.*** The PMLSs at both the pNAR and the NMP detect the movement of the NMP. These nodes employ the link-layer indication sent from the link-layer interface to detect the movement of the NMP [29]. The link-layer triggers alert the network layer module to the status of the link (link up/down). After detecting the movement of the NMP using this link-layer indication, the PMLS at the pNAR starts buffering for the newly arrived Interests. If the pNAR does not receives Face Update message from NMP for a certain period of time as the NMP suddenly goes down, it drops the Interest and deregister the corresponding content using the routing protocol.

***Step 3.*** The NMP changes its point of attachment to a new NAR (nNAR).

***Step 4.***

- The NMP and nNAR create new NDN faces to connect to each other. The APP at the NMP then registers the content name prefixes to the nNAR.
- The PMLS at the NMP re-establishes the connection between itself and pNAR by sending a “Face update” message to the peer PMLS at the pNAR. Upon receiving this, the pNAR could know the new transport end point information of the NMP through the address information field in the Face Update message. This allows the pNAR to know in which NAR the current NMP is located.

***Step 5.***

- The PMLS at the pNAR re-synchronizes the transaction states with the peer PMLS at the NMP by sending a Status Report message per an instance of the transaction structure. The PMLS at the NMP checks whether it has missed any sent Interests before it moves. If any missed Interests exist, it demands re-transmission of the missed Interest to the pNAR. It also checks the last “Data Segment” message received by the pNAR if any. The NMP immediately transmits the remaining data segments. If any buffered Interests exit from the PMLS of the pNAR, they are forwarded to the NMP.
- The NLSR at new NAR advertises newly registered content name prefixes.

***Step 6.*** When the convergence timer at the NMP’s PMLS expires and it does not receive a Status Report message from the NAR for a certain period of time, it sends the “Face Deletion” message to the pNAR to request the deletion of the Face. If there are no more pending Interests in the pNAR’s PMLS, the NLSR at the pNAR withdraws the context name prefixes registered by the NMP’s APP operation.

***Step 7.*** After the convergence timer for the de-registration of the content name prefixes has expired, the old NDN faces connecting the NMP with the pNAR delete themselves.

## 6. Analytical Investigation

In this section, we compare the original NDN handover procedure [3] with the proposed PMLS in terms of the handover cost.

### 6.1. Cost Modeling

An analytical cost model based on [30] is used to compare the handover cost. The handover cost $C_{S}^{\left(\cdot \right)}$ is calculated by adding the additional signaling overhead that results from transmitting handover-related messages and the cost of sending an existing transaction. Since the mobility of NMP is not reflected in these formulas, the mobility model is considered to represent the movement of the NDN producer. We assume that all NARs have a circular coverage with radius R. The NAR’s coverage (denoted by S) is expressed as $S=\pi *R^{2}$. The NMP moves with velocity *v*. Therefore, the NAR crossing rate of the NMP (denoted by $\mu_{a}$) is represented as $2*v/\sqrt{\pi *S}$. The handover cost to which the mobility model is applied can be expressed as $\mu_{a}\cdot C_{S}^{\cdot }$. And the notations used for cost analysis are set with reference to [31]. Table 1 summarizes the definitions of the parameters used for the analytical investigation.

#### 6.1.1. Generic Link Service

When a handover of NMP occurs on an existing NDN, the entire data packet should be re-transmitted, even if the consumer has already received part of the data. When data retransmission is required, the data packet should be segmented into N data segments by the generic link service. Therefore, the handover cost of the generic link service is the sum of the cost of transmitting interest from the consumer to the NMP and the cost of retransmitting the M data segments from the NMP to the consumer. The hop distance from consumer to pNAR and weight of wire and wireless link were reflected to the formula. Therefore, the handover cost of generic link service applying the NAR crossing rate of the NMP is expressed as

(1) $$\begin{array}{cc} C_{S}^{\left(Generic\right)}= & \mu_{a}\{\left(P_{Int}+N*M_{Data}\right)\\ \; & \;\;*(H_{Hop}*\alpha +H_{Wireless}*\beta )\} \end{array}$$

#### 6.1.2. Producer Mobility Link Service

When an NMP moves to a new NAR’s coverage, it restores the connection with the pNAR by sending a face update message to the peer PLMS at the pNAR. Then, it re-synchronizes the transaction states with the peer PMLS by sending a status report message per transaction. Therefore, the handover cost of the PLMS is the sum of the cost of transmitting these control message between pNAR and NMP and the cost of transmitting the (N-K) data segments from the NMP to the pNAR. K means the number of segments that have already been transmitted to the pNAR. As the PLMS can retrieve only the lost data segments using the status report message from pNAR, it does not need to send entire transaction. The hop distance from nNAR to pNAR and weight of wire and wireless link were reflected to the formula. This process allows the NMP to retransmit the portion of the entire data that had not been transmitted to the pNAR. Therefore, the handover overhead of the PLMS applying the NAR crossing rate of the NMP can be represented as

(2) $$\begin{array}{cc} C_{S}^{\left(PMLS\right)}= & \mu_{a}\{\left(M_{Update}+M_{Status}\right)\\ \; & \;*(H_{PtoN}*\alpha +H_{Wireless}*\beta )\}\\ \; & +\mu_{a}\{\left(\left(N-K\right)*M_{Data}\right)\\ \; & \;*(H_{PtoN}*\alpha +H_{Wireless}*\beta )\} \end{array}$$

The default parameter values are set as follows: $P_{Int}$ = 44 bytes, $M_{Update}$ = 28 bytes, $M_{Status}$ = 31 bytes, $P_{Data}$ = 1 MB, $M_{Data}$ = 1033 bytes, and N = 1024, according to [18,32,33,34]. In addition, α is set to 1, β is set to 1.5, and R is set to 400 m, based on [35]. We set the value of $H_{Wireless}$ to 1 and the value of $H_{PtoN}$ to 1 according to the simulation topology.

**Figure 5:**
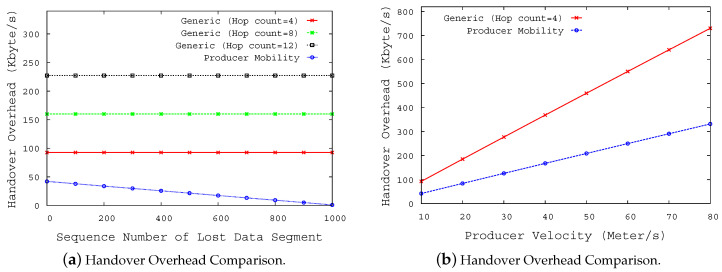
NDN packet sequence at NDN producer and consumer.center

Figure 5a shows the handover overhead of original NDN and the PLMS according to parameter K and $H_{Hop}$ from the consumer and a new NAR. The handover overhead of the original NDN is always greater than that of PMLS. This is because the consumer in the original NDN mobility scheme must request entire transaction, even if it has already received part of the transaction, whereas the PLMS can retrieve only the lost data segments using the status report message. Therefore, the handover cost of the PLMS decreases as the more segments have already been transmitted to the pNAR. In other words, the smaller the number of segments to be sent after handover, the lower the handover cost. On the other hand, in the original NDN, as the consumer must regenerate an Interest packet even though part of the data segment has already received, the handover cost of the original NDN is proportional to the number of hops ($H_{Hop}$) from the consumer to the new NAR. Also, the larger the total size of data which the producer must send back to the consumer, the greater the handover cost of the original NDN would also increase. By contrast, in PLMS, the handover operation occurs only between the producer and the previous NAR, so the handover overhead is constant regardless of $H_{Hop}$.

Figure 5b shows the handover overhead versus velocity (v). In original NDN mobility and PLMS, the handover overhead increases in direct proportion to increases in the vehicle speed, but the handover overhead of the original NDN mobility is higher than that of PLMS. This is because the signaling required for handover is limited to 1 hop in the case of PLMS, while the signaling required for original NDN mobility increases according to the number of hops between the consumer and the new NAR.

## 7. Simulation Investigation

### 7.1. Simulation Environment

**Figure 6:**
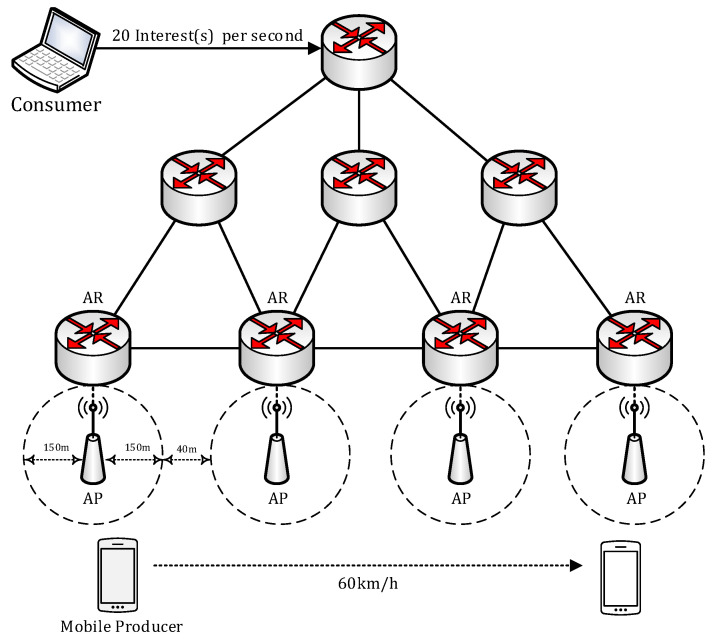
Simulation Network Topology.

We used a modification of the ndnSIM 2.3 [36], an open source NS-3-based NDN simulator, to assess the handover performance of our proposed scheme. The PMLS is a subclass of the generic link service, so it provides all the services supported by the generic link service. It also handles the PMLS control messages to support link recovery. The ndnSIM 2.3 does not support any name-based routing protocol. We updated the FIB tables at the NDN routers within the network after the average OSPF convergence time (6 s) [37] has elapsed, since the NLSR, which is the default NDN routing protocol, is derived from the OSPF routing protocol. Figure 6 shows the network model used in this simulation. The NDN network uses the IPv4 network as its transport network. We assumed that the transport network supports the use of anycast-style addresses that route messages from consumer to the nearest NAR. This can be archived by statically installing anycast-specific route information to the default access routers of the IPv4 network. Each NAR is connected to an Access Point (AP) covers an area with a 150 m radius. The distance between two APs is 340 m, so there is a 40 m shadow area between two APs. The NMP moves to 60 km per hour with the constant velocity mobility model from AP 1 to AP 2 direction. Upon connecting to a new NAR (nNAR),), the NMP registers its content name prefixes before it receives the first Interest from the nNAR. The consumer sends 20 Interests per second (1 Interest every 50 ms). The hop count between entities is set to 1 and the link delay for each hop is set to 10 ms.

### 7.2. Simulation Results

**Figure 7:**
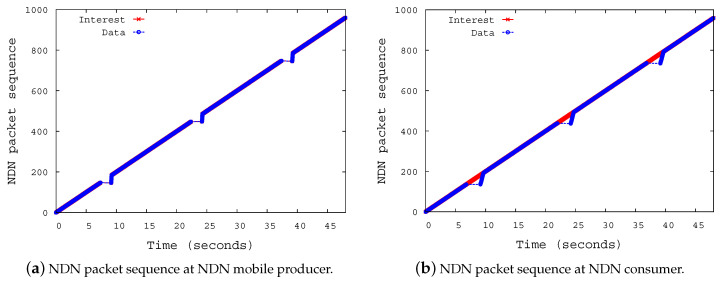
NDN packet sequence at NDN producer and consumer.center

In the simulation investigation, we measured the NDN packet (Interest, Data) sequence in an effort to prove that the proposed scheme could prevent the loss of Interests even in the occurrence of a handover of the NMP. Figure 7a shows the Interest reception sequence at the NMP. The NMP receives the 140th Interest at 7.02015 s and it receives the 141st Interest at 9.46955. The figure shows that no loss of Interest occurred even though NMP moved from AP 1 to AP 2. There is a gap of 2.4494 s between the reception of the two Interests. The NMP takes 2.4 s to move out of the shadow area (40 m). The NMP and nNAR have a 49.5 ms interaction. In this time, they can exchange four control messages, as the link delay between them is 10 ms. This process is repeated each time the NMP encounters a new AP, as shown in Figure 7a.

The NDN packet sequence at the consumer is shown in Figure 7b. The 140th and the 141st Data packets are delivered at 7.05008 s and at 9.50909 s after the handover of the NMP has occurred, respectively. The NDN consumer sends the 141st Interest at 7.05 s and it receives the corresponding 141st Data packet at 9.50909 s. The delay is 2.459 s, which is less than the default Interest lifetime (4 s). Although there is a time when the consumer does not receive Data for each time there is a handover event of the NMP, seamless Data delivery is always guaranteed to the consumer.

**Figure 8:**
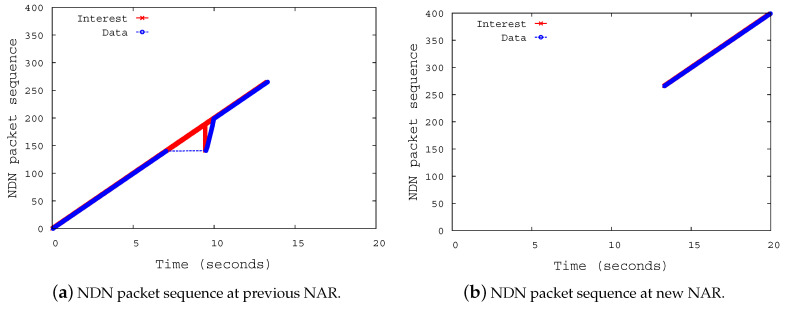
NDN packet sequence at NDN routers.center

Figure 8 shows the NDN packet sequence at the NDN routers. Shown in Figure 8a, the pNAR continues to receive Interests from the consumer even though the NMP has moved to nNAR. After recovering the broken link with the NMP, the pNAR sends the buffered Interests to the NMP. Furthermore, it can be confirmed that the NDN data packets sent from the NMP are delivered to the consumer via pNAR until the routing information by the NLSR is propagated over the network.

After the average OSPF convergence time (6 s) has elapsed since the NMP has attached at nNAR, the NAR at which the NDN consumer is attached updates its FIB table. Figure 8b shows that the Interests sent by the consumer are transferred to the nNAR at which the NMP is attached, and that the Data packets corresponding to the Interest are also transferred to the consumer via the nNAR.

**Figure 9:**
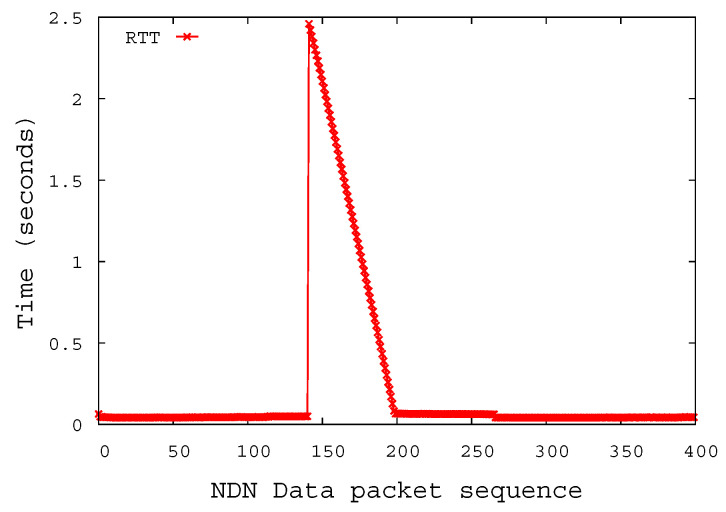
Round-trip time for NDN packets at NDN consumer.

Figure 9 shows the Round-Trip Time (RTT) between the consumer sending the Interest packet and receiving the Data packet. The RTT increases up to 2.45909 s by the time the NMP is in the shadow area. As the NMP moves toward the center of the nNAR, the RTT decreases continuously. After the routing information is updated by the NLSR, the data packet reaches the consumer via the nNAR, not the pNAR. This was confirmed by the difference between the RTT of the 265th NDN packet and the RTT of the 266th NDN packet; the 265th and 266th Interests are forwarded to different NARs. The consumer sees that the RTT between sending the Interest and receiving the Data packet is reduced by 20 ms from 60 ms to 40 ms. This is due to the facts that the number of hops the data packet goes through is reduced by two and that each link delay is 10 ms.

**Figure 10:**
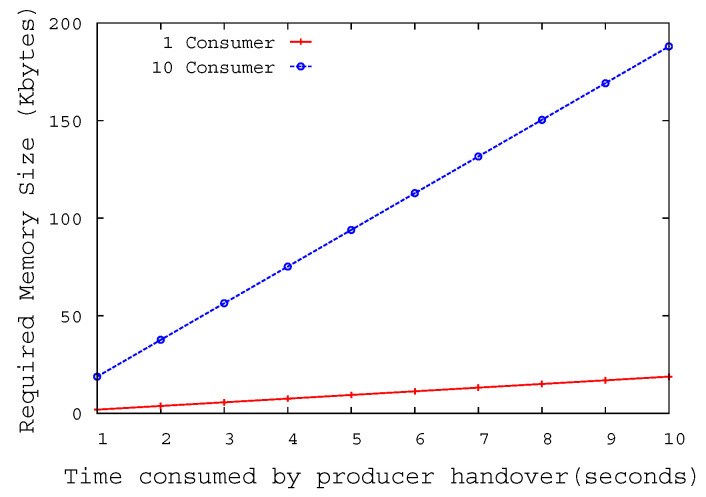
Required buffer size at NAR.

If the core router to have optimal performance under the constraint that the router’s buffer size must be small, the buffer size of the router must be greater than or equal to 2T ∗ C ÷$\sqrt{N}$ [38]. 2T means the Round-Trip Time between producer and consumer, and C stands for bottleneck link capacity between the router and producer. N is the number of flows which share a bottleneck link. We assumed that the capacity of the wired link is 1 Gbps and has a delay of 1 ms, and total 10 consumers send interest to the producer. According to the calculation in the simulation environment, the ideal buffer size required for NAR is about 316K bytes. Figure 10 shows the size of the required buffer according to the time it takes for the NMP to restore the connection with pNAR after the link down event occurs. When the pNAR must buffer the interests from 10 consumers for 10 s, the buffer size required for pNAR is 188K byte. Since the optimal buffer size calculated above is 316K bytes, pNAR could additionally buffer interests for about 20 s. However, as the pNAR cannot buffer interests indefinitely, it is necessary to choose a default value to determine how long to buffer the interests considering the distance between NARs.

## 8. Discussion

In the future, we plan to investigate the actual implementation of the proposed PMLS by using embedded devices such as Raspberry Pi or Arduino. For this practical implementation, we will use an open NDN protocol stack called Named Data Networking Forwarding Daemon (NFD). However, since NFD is implemented as a general computer target, additional porting work will be required to operate NFD in the above embedded devices. In addition, we will apply the proposed producer mobility link service to the NDN protocol stack. Through this practical implementation, we will verify whether the proposed PMLS is applicable in real environments. In addition, we plan to research a solution that can support producer mobility more robustly even when mobile producers move very quickly and randomly and to prove detailed performance analysis with existing anchorless methods.

## 9. Conclusions

We propose a producer mobility link service with which to enhance the producer mobility service under the NDN architecture. It attempts to extend the period in which the forwarding path to the producer is relevant by concealing link breakage due to the producer movement to the NDN system. The proposed scheme repairs the broken link by re-establishing a new connection between the NMP and the previous NAR, and then recovering the disrupted on-going transmission. In order to recover the disrupted on-going transmission between the NMP and the previous NAR, it treats the exchange of the <Interest, Data Packet> pair as a transaction. Please note that the proposed producer mobility scheme is transparent to the rest of NDN architecture, as it is in an NDN face. The simulation investigation results indicate that the proposed scheme is practical and effective for the NDN producer mobility solution.

## Figures and Tables

**Table 1:** Definition of parameters required for analytical Investigation.

Parameter	Comments
$P_{Data}$	Data packet for the interest packets
$P_{Int}$	Interest packet to request data
$M_{Data}$	Data segment in which a data is divided into N segments
$M_{Update}$	Face update Message from the NMP
$M_{Status}$	Status report message from the previous NAR
*N*	Total number of segmented data messages
*K*	The sequence number of the first missing segment
$H_{Hop}$	Hop count from consumer to new NAR
$H_{PtoN}$	Hop count from previous AR to new NAR
$H_{Wireless}$	Hop count from AP to producer
α	Weights of the wire link
β	Weights of the wireless link
